# Association study of *SHANK3 *gene polymorphisms with autism in Chinese Han population

**DOI:** 10.1186/1471-2350-10-61

**Published:** 2009-06-30

**Authors:** Jian Qin, Meixiang Jia, Lifang Wang, Tianlan Lu, Yan Ruan, Jing Liu, Yanqing Guo, Jishui Zhang, Xiaoling Yang, Weihua Yue, Dai Zhang

**Affiliations:** 1 Institute of Mental Health, Peking University, Beijing, PR China; 2 Key Laboratory for Mental Health, Ministry of Health, Beijing, PR China; 3 Beijing Children's Hospital Affiliated to Capital University of Medical Sciences, Beijing, PR China

## Abstract

**Background:**

Autism, a heterogeneous disease, is described as a genetic psychiatry disorder. Recently, abnormalities at the synapse are supposed to be important for the etiology of autism.*SHANK*3 (SH3 and multiple ankyrin repeat domains protein) gene encodes a master synaptic scaffolding protein at postsynaptic density (PSD) of excitatory synapse. Rare mutations and copy number variation (CNV) evidence suggested *SHANK3 *as a strong candidate gene for the pathogenesis of autism.

**Methods:**

We performed an association study between *SHANK3 *gene polymorphisms and autism in Chinese Han population. We analyzed the association between five single nucleotide polymorphisms (SNPs) of the *SHANK3 *gene and autism in 305 Chinese Han trios, using the family based association test (FBAT). Linkage disequilibrium (LD) analysis showed the presence of LD between pairwise markers across the locus. We also performed mutation screening for the rare de novo mutations reported previously.

**Results:**

No significant evidence between any SNPs of *SHANK3 *and autism was observed. We did not detect any mutations described previously in our cohort.

**Conclusion:**

We suggest that *SHANK3 *might not represent a major susceptibility gene for autism in Chinese Han population.

## Background

Autism is a pervasive developmental disorder mainly characterized by limited or absent verbal communication, lacking of reciprocal social interaction or responsiveness and restricted, stereotypical, and ritualized patterns of interests and behavior. Autism together with childhood disintegrative disorder, pervasive not otherwise specified (PDD-NOS, or atypical autism) and Asperger syndrome share the similar characteristics and are all included as autism spectrum disorder (ASD), also known as pervasive developmental disorder (PDD). Family and twin studies have conclusively described autism as a highly heritable neuropsychiatry disorder with heritability estimates of over 90% and the environmental factors contributing no more than 10% [1-3]. Nevertheless, autism is etiologically heterogeneous.

Shank3 (SH3 and multiple ankyrin repeat domains 3; also termed ProSAP2, proline-rich synapse-associated protein 2) is a master synaptic scaffolding protein[4,5]. In rats and human beings, Shank3 is expressed preferentially in cerebral cortex and cerebellum[6,7]. With its multiple protein interaction domains, this molecule directly or indirectly connects with neurotransmitter receptors and cytoskeleton proteins[8,9]. It also participates in the formation, maturation and enlargement of dendritic spines and is essential for the formation of functional synapses[10].

Accumulating discoveries indicate that autism's cause may reside in abnormalities at the synapse[3]. Synapses are the physical sites through which neurons in the brain connect with each other into an integrated circuit. In 2003, the alterations in synaptic function was first proposed to be a possible cause of autism[11]. Neuroligins are a family of postsynaptic cell adhesion molecules and may be involved in the synaptogenesis[12]. Mutations of genes encoding neuroligins (NLGN3, NLGN4X) were supposed to be pathogenic for autism and Asperger syndrome[13]. Neuroligin-deficiency mouse models according to these findings exhibit some deficits that are reminiscent of ASD in human[14,15]. The "neuroligin autim pathway" was postulated[3]. Shank3 acts as a binding partner for neuroligins(NLGNs)[16]. It was reported that rare mutations in *SHANK3 *may contribute to the pathogenesis of autism. Durand et al. reported two de novo alterations in *SHANK3 *in subjects with ASD but not in control individuals. One is a G insertion, and the other is a deletion of the terminal 22q13 with the breakpoint in intron8 of *SHANK3*[17]. Moessner et al. identified de novo variants with an A962G exchange in exon8 leading to a heterozygous Q321R substitution[18]. They also reported a heterozygous deletion encompassing *SHANK3 *in a female proband but in neither parent nor in two unaffected brothers[18]. Recently Gauthier et al. found a de novo deletion at an intronic splice site in their autistic patients, and this deletion will lead to aberrant splicing of the transcript[19]. *SHANK3 *could also belong to the "NLGN autism pathway"[1].

All these indicate that *SHANK3 *might be a strong candidate gene for autism. Resequencing has been applied to identify the rare mutations of this gene, but the linkage and association studies of *SHANK*3 are still insufficient. In this study, we attempted to investigate the association between the *SHANK3 *gene polymorphisms and autism in 305 Chinese Han trios on a population-based approach using the family-based association test (FBAT). We also performed mutation screen of this gene in probands with autism in order to detect the rare de novo mutations reported previously.

## Methods

### Subjects

The sample for this study consisted of 305 Chinese Han family trios (singleton autistic disorder patients and their unaffected biological parents). These families were recruited at the Institute of Mental Health, Peking University, China. Of the 305 autistic child probands, 281 were male and 24 were female. The mean age of the children at the time of testing was 11 years (range 3–25 years). Diagnoses of autism were established by senior psychiatrists. All patients fulfilled the DSM-IV criteria for autistic disorder. The cases were assessed using childhood autism rating scale (CARS)[20] and autism behavior checklist (ABC)[21]. Children with fragile × syndrome, tuberous sclerosis, a previously identified chromosomal abnormality, dysmorphic features, or any other neurological condition suspected to be associated with autism were excluded. All subjects provided written informed consent for participation in this study. The study was approved by the Ethics Committee of the Health Science Center, Peking University.

### Genotyping and sequencing

Genomic DNA was extracted from the blood using a Qiagen QIAamp DNA Mini Kit. We selected seven single nucleotide polymorphisms (SNPs) in the *SHANK3 *gene according to the dbSNP  and the international HapMap project , including rs9616915(missense polymorphism), rs2106112(synonymous polymorphism), rs6010065, rs13057681(missense polymorphism), rs2301584(3'UTR), rs41281537 and rs756638. Mutation screen for the rare de novo mutataion reported previously was performed by sequencing exon8 (for A962G[18]), exon21 (for G insertion[17]) and donor splice site G deletion of intron19[19] in all the 305 probands with autism. The SNPs with frequencies of minor allele frequency (MAF) in our sample greater than 5% were used as genetic markers in this study. Three SNPs (rs9616915, rs6010065, rs13057681) were analyzed by polymerase chain reaction-restriction fragment length polymorphism (PCR-RFLP) analysis. Direct DNA sequencing was used for analyzing the other SNPs (rs2106112, rs2301584, rs41281537, rs756638) and detecting the rare de novo mutation reported previously. The information of primers and PCR-RFLP analysis is given in Table 1. The PCR amplification was performed in a 25 μl volume containing GC Buffer (TaKaRa), 200 mM of each dNTPs, 0.3 mM of each primer, 1 U of Taq DNA polymerase, and 40 ng of the genomic DNA. The conditions used for PCR amplification were an initial denaturation phase at 94°C for 5 min, followed by 36 cycles at 94°C for 30 sec, annealing at 60–65°C for 30–60 sec, and extension at 72°C for 30 sec, followed by a final extension phase at 72°C for 7 min. A 15 μL aliquot of the PCR product mixtures was completely digested with 3 units of restriction enzyme overnight. Digestion products were visualized through ethidium bromide staining after electrophoresis in 1%–3% agarose gels. The DNA sequencing was performed after cleaning the PCR product using a BigDye Terminator Cycle Sequencing Ready Reaction Kit with Ampli Taq DNA polymerase (PE Biosystem). The inner primers were used for the cycle-sequencing reaction, and the fragments were separated by electrophoresis on an ABI PRISM 377-96 DNA Sequencer (Applied Biosystem, Foster city, U.S.A).

**Table 1:** Information of the primers and PCR-RFLP Analysis

SNP	Primer sequence (5'→3')	Product (bp)	RFLP	Allele (bp)	
rs9616915	Forward: acctgggggcttcacctgacta Reverse: cccaacccagcacacagagg	207	*Ava *II	T (180/27)	C (121/59/27)
rs13057681	Forward: ccgcaagagccccctggtga Reverse: caggaggcgctcgtcgatggag	328	*Sty *I	G (198/130)	C (328)
rs6010065	Forward: ccttacctgggtgggcatt Reverse: acggggagggctcttgtg	423	*Hinf *I	G (166/257)	C (423)
rs2106112	Forward: ctgagccactcggaggttgct Reverse: gtccgaccttcaccacgttcac	340			
rs2301584, rs41281537, rs756638	Forward: cgccaacagtccaggtcac Reverse: cagaaggcatgggctgagtt	489			
G insertion in exon21	Forward: cgggaggagcggaagtcac Reverse: ggaccccgttggcaaactct	409			
A962G in exon8	Forward: cgcacgccatgtgtgcattcct Reverse: tctcggggttggggggtcagac	473			
splice doner site of intron19	Forward: gcctggggtggggtctgag Reverse: ttggagcctgggctgtgtg	754			

PCR-RFLP, polymerase chain reaction-restriction fragment length polymorphism; SNP, single nucleotide polymorphism.

### Statistical analyses

Deviation from the Hardy-Weinberg equilibrium (HWE) for genotype frequency distributions was analyzed using the Chi-square goodness-of-fit test. To perform single- and multi-locus tests of association, we used the FBAT program (v. 1.5.1)[22]. The FBAT program uses a generalized score statistic to perform a variety of transmission disequilibrium tests, including haplotype analysis. Moreover, the FBAT program provides pairwise linkage disequilibrium (LD) analysis to detect an inter-marker relationship, using *D' *values. SNP pairs were considered to be in strong LD if *D' *> 0.70. The global haplotype tests of association were performed under "multiallelic" mode in haplotype FBAT. Meanwhile, the individual haplotype tests were conducted under "biallelic" mode in haplotype FBAT. Family-based association tests were performed under an additive model in the present study. The significance level for all statistical tests was two-tailed *P *< 0.05.

## Results

Seven SNPs in *SHANK3 *gene were genotyped in 305 Chinese Han autism trios. SNPs rs2106112 and rs13057681 were not polymorphic in our samples. They were not used as genetic markers for the association analyses. The other five SNPs (rs9616915, rs6010065, rs2301584, rs41281537, rs756638) were polymorphic with MAF > 5% and were then used as genetic markers for the association study. None of the genotype distributions of these five SNPs in parents or patients deviated from Hardy-Weinberg equilibrium (data not shown). Allele frequencies and the results of FBAT for single SNP analyses are shown in Table 2. None of the five SNPs was significant evidence (*P *< 0.05) for preferential transmission of an allele by FBAT in all samples. To further analyze the pattern of linkage disequilibrium (LD) in our sample, we computed pairwise LD for all possible combination of the five SNPs using *D' *values (Table 3). Four SNPs (rs6010065, rs2301584, rs41281537, rs756638) were found to be in strong LD with each other (*D' *> 0.7) except for rs9616915 (Table 3). To determine whether any specific haplotype would confer a higher risk for autism, we tested all specific and globe haplotype composed of these SNPs. Two haplotypes displayed weak association with autism. These are C-G-G-A (rs6010065- rs2301584- rs41281537- rs756638, *P *= 0.040) and G-A (rs41281537- rs756638, *P *= 0.047), respectively (Table 3). After 1,000 permutation tests the haplotype C-G-G-A was still associated with autism (*P *= 0.041), but no significance remained in the haplotype G-A (*P *= 0.050). No statistically significant association (*P *< 0.05) was observed for any other haplotypes in any set of families in HBAT analyses (data not shown). Moreover, the entire global test for haplotype transmission revealed a negative result (data not shown). To detect the de novo mutations reported before, we sequenced the exon8 (for A962G)[18], part of exon21 (for G insertion)[17], the donor splice site of intron19[19] of *SHANK3 *in 305 probands. However, we didn't find any genetic variants or the mutations reported previously.

**Table 2:** Results of FBAT for the five SNPs

Makers	Allele	Afreq	Families	S	E (S)	Var (S)	Z	*P*-values
rs9616915	T	0.911	87	128.000	123.500	24.750	0.905	0.365
	C	0.089	87	46.000	50.500	24.750	-0.905	0.365
rs6010065	G	0.500	235	242.000	236.500	79.250	0.618	0.536
	C	0.500	235	228.000	233.500	79.250	-0.618	0.536
rs2301584	G	0.811	159	218.000	217.000	46.000	0.147	0.882
	A	0.189	159	100.000	101.000	46.000	-0.147	0.882
rs41281537	G	0.858	133	188.000	189.500	37.750	-0.244	0.807
	A	0.142	133	78.000	76.500	37.750	0.244	0.807
rs756638	G	0.812	155	218.000	213.000	45.500	0.741	0.458
	A	0.188	155	92.000	97.000	45.500	-0.741	0.458

FBAT, family-based association test; Afreq, allelic frequency. Families, number of informative families; S, test statistics for the observed number of transmitted alleles; E(S), expected value of S under the null hypothesis (i.e., no linkage or association). Significant *P *values (< 0.05) are in boldface.

**Table 3:** Measure of Pairwise Linkage Disequilibrium D (*D'*) Between Five SNPs in *SHANK3 *gene

	rs9616915	rs6010065	rs2301584	rs41281537
rs6010065	-0.024(0.58)			
rs2301584	-0.003(0.21)	0.082(0.87)		
rs41281537	-0.010(0.82)	0.068(0.95)	-0.023(0.86)	
rs756638	-0.007(0.44)	0.081(0.86)	-0.027(0.77)	0.115(1.00)

The *SHANK3 *gene spans about 60 kb. In Affimatrix SNP 5.0 chip of 240 trios from this sample, it included 16 CNV probes covering *SHANK3 *gene and its flanking region. The smallest distance between two probes was less than 500 bp. In these probes, one CNV probe was located at intron8 which was reported as a breakpoint of a de novo deletion[17], and a few CNV probes were quite close to exon21 which was reported as a breakpoint of a de novo translocation in 22q13 deletion syndrome[7]. However, we didn't find any genomic imbalance in all these 240 trios or control (unpublished data).

## Discussion

Shank3 is a master synaptic scaffolding protein, acting as the bridge of some neurotransmitter receptors and the downstream signal transduction. It has been postulated to perform important roles in excitatory synapse assembly [23-26], dendritic formation and maturation[10,27,28]. The de novo alterations of this gene and their roles in the pathogenesis of autism have been reported by some studies [17-19]. Based on this evidence, we hypothesized that the *SHANK3 *might be a strong candidate gene for autism. However, there was few evidence of association between *SHANK3 *polymorphisms and autism. In the present study, we investigated the association of *SHANK3 *polymorphisms and autism in 305 Chinese Han trios. We detected seven dbSNPs of *SHANK3*, including two nonpolymorphic SNPs rs2106112 (synonymous mutation) and rs13057681 (H1033D) in our samples. In a family-based association study for all the other five SNPs we found no evidence for transmission disequilibrium for any single marker (*P *> 0.05), even for the missense SNP rs9616915C>T which was reported as a non-synonymous variant identified in ASD[19]. In attempt to identify de novo genetic variants in *SHANK3 *that had been reported previously, we performed mutation screen of exon8, part of exon21, the donor slpice site of intron19 of *SHANK3 *in 305 probands with autism, and only identified one novel synonymous variant (T1231). In addition, the specific and global-haplotype FBAT tests of association were performed. Four SNPs were located in a block with high LD including rs6010065, rs2301584, rs41281537, rs756638. However, only C-G-G-A (rs6010065- rs2301584- rs41281537- rs756638, P = 0.040) exhibited a weak association with autism in our sample. We didn't detect any genomic imbalance of *SHANK3 *and its flanking region in our sample using Affimetrix 5.0 chip either. Our finding suggested that *SHANK3 *doesn't represent a major susceptibility gene for autism in the autism families ascertained from Chinese Han population.

Several potential reasons might explain the difference among various reports about *SHANK3 *gene and autism. One might be the racial and ethnic differences in genotype distribution and association with autism risk. For example, the allele frequency of the *SHANK3 *non-synonymous SNP rs9616915 was obviously different between Chinese and the other populations in the National Center for Biotechnology Information (NCBI) gene database. For European (CEU), and Nigeria population, the frequencies of allele C are 0.533 and 0.383 respectively, but for Chinese Han population (CHB) it is 0.044. Further, the variety of the investigated samples should be noticed. The previous evidence about *SHANK3 *was always from subjects with ASD, including autism, Asperger syndrome and PDD-NOS. Our autism cases only include patients with infantile autism, and not other cases of the etiologically more heterogeneous ASD. The heterogeneity of the investigated samples combined with the heterogeneity of neurodevelopmental disorders might hamper the understanding of genetic factors associated with autism. In order to enhance the possibility of finding relevant genetic cause, the phenotype variability in sample should be reduced[29]. In the present study we examined a relatively homogenous sample of autism to reduce the heterogeneity. The location of *SHANK3 *is at 22q13.3, which is a critical region for 22q13 deletion syndrome that also has autistic behavior. There are strong evidence that haploinsufficiency of *SHANK3 *plays a major role in 22q13 deletion syndrome[30]. *SHANK3 *is very likely to be involved in the pathogenesis of some mutual phenotypes of ASD and 22q13 deletion syndrome, such as delay of expressive speech. Furthermore, 22q13 deletion syndrome has a clinical phenotype overlapping in part the ASD phenotype. So subjects with 22q13 deletion may be included in ASD samples. It is really critical for the researches on autism to exclude the 22q13 deletion syndrome. We didn't detect any genomic imbalance of 22q13 in our samples using Affimetrix SNP 5.0 chip. Moreover, although the disruption of *SHANK3 *seems to be associated with 22q13 deletion syndrome, there is also contrary evidence that the haploinsufficiency for 22q13 genes other than *SHANK3 *have major effects[31]. The present study indicates that *SHANK3 *may not be a critical gene for the etiology of infantile autism in Chinese Han population. As autism is a heterogeneous disease, the rare mutations of *SHANK3 *gene seem to explain the etiology of only a small proportion of cases with autism. Sykes et al. reported recently that they didn't find any CNV or SNP association of *SHANK3 *within their ASD sample, although they didn't sequence the gene[32]. Their suggestion that *SHANK3 *deletions may be limited to a portion of autism was coincident with ours.

There was few association or linkage study for *SHANK3 *and autism. Our family-based association study provided an indication that *SHANK3 *was not critical for the pathogenesis of autism in Chinese Han population or only account for a small proportion of autism individuals. In addition, our results also reinforce the need for the detailed LD mapping, mutation screening and CNV analysis of *SHANK3 *in different population or other neurodevelopmental disorders.

## Conclusion

The present study did not find strong evidence of *SHANK3 *polymorphisms and autism or identify any described non-synonymous mutations in our cohort. These might indicate that *SHANK3 *doesn't represent a major susceptibility gene for autism in the autism families ascertained from Chinese Han population.

## Competing interests

The authors declare that they have no competing interests.

## Authors' contributions

JQ carried out sequencing and PCR-RFLP analysis, and contributed to manuscript writing. MJ performed the statistical analysis and most of the sample collection. LW aided in statistical analysis. TL and YR aided in sample storage and preparations. JL, YG, JZ and XY contributed to the sample collection. WY and DZ participated in the design of the study and manuscript writing.

## Pre-publication history

The pre-publication history for this paper can be accessed here:


